# Supplementary material for: Comparative Gene Expression Profiles in Parathyroid Adenoma and Normal Parathyroid Tissue
Source: J Clin Med. 2019 Mar 2;8(3):297. doi: 10.3390/jcm8030297 (PMC6463127; doi:10.3390/jcm8030297)
Supplement: Supplementary file 1 [file jcm-08-00297-s001.zip › Supplemental Table 3. All GO terms for down-regulated genes.docx]

Supplemental Table 3. GO terms and KEGG pathways for down-regulated genes

| **Category** | **TermID** | **Term** | **Count** | **PValue** | **Genes** |
| --- | --- | --- | --- | --- | --- |
| GOTERM_BP_DIRECT | GO:0002474 | antigen processing and presentation of peptide antigen via MHC class I | 5 | 2.81E-04 | CANX,SEC13,PDIA3,CALR,BCAP31 |
| GOTERM_BP_DIRECT | GO:0061077 | chaperone-mediated protein folding | 5 | 6.40E-04 | FKBP9,CANX,PEX19,PPIB,CALR |
| GOTERM_BP_DIRECT | GO:0006465 | signal peptide processing | 4 | 0.002342 | SPCS2,SPPL2A,SEC11A,SEC11C |
| GOTERM_BP_DIRECT | GO:0006506 | GPI anchor biosynthetic process | 4 | 0.002627 | PIGU,ALG5,PIGG,PIGP |
| GOTERM_BP_DIRECT | GO:0044829 | positive regulation by host of viral genome replication | 3 | 0.003067 | YTHDC2,VAPB,PPIB |
| GOTERM_BP_DIRECT | GO:0036498 | IRE1-mediated unfolded protein response | 5 | 0.003672 | SEC63,XBP1,SEC61G,PDIA6,HYOU1 |
| GOTERM_BP_DIRECT | GO:0002576 | platelet degranulation | 6 | 0.00503 | LAMP2,APLP2,LGALS3BP,CD36,FAM3C,PSAP |
| GOTERM_BP_DIRECT | GO:0006888 | ER to Golgi vesicle-mediated transport | 7 | 0.007614 | TMED2,RAB2A,VAPB,RAB1A,HYOU1,TMED10,BCAP31 |
| GOTERM_BP_DIRECT | GO:0034975 | protein folding in endoplasmic reticulum | 3 | 0.008247 | CANX,PDIA3,CALR |
| GOTERM_BP_DIRECT | GO:0018279 | protein N-linked glycosylation via asparagine | 4 | 0.008947 | UGGT2,ALG5,STT3A,RPN1 |
| GOTERM_BP_DIRECT | GO:0098609 | cell-cell adhesion | 9 | 0.009027 | CAPZB,EIF3E,DDX6,EPCAM,VAPB,EIF2S3,RAB1A,BSG,EEF1G |
| GOTERM_BP_DIRECT | GO:0050900 | leukocyte migration | 6 | 0.010131 | CD74,SLC7A8,CD58,MSN,BSG,CD34 |
| GOTERM_BP_DIRECT | GO:0008543 | fibroblast growth factor receptor signaling pathway | 5 | 0.011676 | KL,HNRNPA1,TIAL1,POLR2H,FAT4 |
| GOTERM_BP_DIRECT | GO:0006890 | retrograde vesicle-mediated transport, Golgi to ER | 5 | 0.011676 | TMED2,ERGIC2,RAB1A,TMED10,RAB6A |
| GOTERM_BP_DIRECT | GO:0006457 | protein folding | 7 | 0.013103 | DNAJA1,ST13,FKBP9,CANX,PDIA6,PDIA3,CALR |
| GOTERM_BP_DIRECT | GO:0006413 | translational initiation | 6 | 0.016079 | EIF3E,RPS25,RPL23,EIF2S3,FAU,RPL26 |
| GOTERM_BP_DIRECT | GO:0006614 | SRP-dependent cotranslational protein targeting to membrane | 5 | 0.018453 | SEC63,RPS25,RPL23,FAU,RPL26 |
| GOTERM_BP_DIRECT | GO:0006509 | membrane protein ectodomain proteolysis | 3 | 0.022933 | SPPL2A,NCSTN,ADAM9 |
| GOTERM_BP_DIRECT | GO:0030433 | ER-associated ubiquitin-dependent protein catabolic process | 4 | 0.026538 | ERLIN2,PSMC2,TMEM67,RNF103 |
| GOTERM_BP_DIRECT | GO:0048208 | COPII vesicle coating | 4 | 0.027696 | TMED2,RAB1A,SEC13,TMED10 |
| GOTERM_BP_DIRECT | GO:1903298 | negative regulation of hypoxia-induced intrinsic apoptotic signaling pathway | 2 | 0.031817 | TMBIM6,HYOU1 |
| GOTERM_BP_DIRECT | GO:0019083 | viral transcription | 5 | 0.032461 | RPS25,RPL23,FAU,NUP205,RPL26 |
| GOTERM_BP_DIRECT | GO:0000184 | nuclear-transcribed mRNA catabolic process, nonsense-mediated decay | 5 | 0.039215 | EIF3E,RPS25,RPL23,FAU,RPL26 |
| GOTERM_BP_DIRECT | GO:0031647 | regulation of protein stability | 4 | 0.039311 | LAMP2,XBP1,FBXO7,SUMO1 |
| GOTERM_BP_DIRECT | GO:0006611 | protein export from nucleus | 3 | 0.04085 | XPO1,CSE1L,CALR |
| GOTERM_BP_DIRECT | GO:0006886 | intracellular protein transport | 7 | 0.041964 | CD74,XPO1,TMED2,AP3B1,SEC13,TMED10,BCAP31 |
| GOTERM_BP_DIRECT | GO:0048205 | COPI coating of Golgi vesicle | 2 | 0.042197 | TMED2,TMED10 |
| GOTERM_BP_DIRECT | GO:0016032 | viral process | 8 | 0.042492 | XPO1,SUMO1,IL6ST,HNRNPA1,NUP205,PSMC2,LMBRD1,  RAB6A |
| GOTERM_BP_DIRECT | GO:0007030 | Golgi organization | 4 | 0.045149 | TMED2,RAB2A,RAB1A,TMED10 |
| GOTERM_BP_DIRECT | GO:0034976 | response to endoplasmic reticulum stress | 4 | 0.046672 | XBP1,PDIA6,HYOU1,PDIA3 |
| GOTERM_BP_DIRECT | GO:0045454 | cell redox homeostasis | 4 | 0.049793 | PRDX4,PDIA6,PDIA3,TMX2 |
| GOTERM_BP_DIRECT | GO:0001843 | neural tube closure | 4 | 0.049793 | TMED2,MTHFD1,FZD6,SPINT2 |
| GOTERM_CC_DIRECT | GO:0070062 | extracellular exosome | 79 | 1.29E-17 | LAMP2,CD74,VPS13D,PLPP1,SLC44A1,CAPZB,ITM2A,APLP2,DNAJA1,SLC7A8,RAB18,ST13,MTHFD1,PLP2,RAB2A,EIF3E,  NUCB1,OGN,BLMH,LGALS3BP,CPE,SIAE,SERINC1,UXS1,  PCYOX1,CD58,RPS25,EPCAM,PRDX4,CSE1L,TM9SF2,RPL23,  CANX,VPS13C,PSMA1,EIF2S3,TPT1,KL,IL6ST,HNRNPA1,  ITM2B,RAB1A,SPPL2A,TSPAN3,SEC11A,PDIA6,MSN,ERLIN2,GSN,HYOU1,TIAL1,GJA1,UTRN,SEC13,RPL26,NCSTN,ATP1A1,PPIB,PDIA3,ADAM9,PDCD6IP,TMED10,NUDT9,CLSTN1,BSG,ADGRG2,RAB6A,MYO1D,FUCA1,CALR,PPA1,ATP6AP2,  PTP4A2,APOD,FAT4,FAM3C,IGF2R,PSAP,EEF1G |
| GOTERM_CC_DIRECT | GO:0016020 | membrane | 64 | 2.20E-14 | SLC7A2,LAMP2,CD74,SEC63,IFNGR1,PLPP1,SLC44A1,CAPZB,XPO1,APLP2,AK6,DNAJA1,ERGIC2,SLC15A1,SLC7A8,MTHFD1,PIGU,STAG2,PLP2,EIF3E,NUCB1,LGALS3BP,PRKAR1A,DDX6,CD58,ALG5,P4HA1,CSE1L,RPL23,  CANX,SEC61G,AP3B1,IL6ST,STT3A,CD36,HNRNPA1,ITM2B,  DDX56,YME1L1,SPPL2A,ESYT1,TMBIM6,SCCPDH,DEGS1,  HYOU1,UTRN,NUP205,PSMC2,RPL26,NCSTN,ATP1A1,RPN1,DNAJB14,PPIB,LMBRD1,PDCD6IP,BSG,PIGG,RAB6A,CALR,  BCAP31,FAT4,IGF2R,EEF1G |
| GOTERM_CC_DIRECT | GO:0005789 | endoplasmic reticulum membrane | 36 | 4.40E-12 | SEC63,TMED2,ERGIC2,RAB18,XBP1,PIGU,PLP2,RAB2A,  SERINC1,SPCS2,ALG5,FKBP9,VAPB,CANX,SEC61G,STT3A  ,RAB1A,TMBIM6,SEC11A,DEGS1,PDIA6,ERLIN2,GJA1,SEC13,  RPN1,DNAJB14,TMEM67,SEC11C,FAXDC2,TMED10,CLSTN1,PIGG,RAB6A,PIGP,BCAP31,RNF103 |
| GOTERM_CC_DIRECT | GO:0005783 | endoplasmic reticulum | 31 | 2.70E-09 | SEC63,IFNGR1,YTHDC2,TMED2,XBP1,PLP2,UGGT2,OGN,FKBP9,NT5C3A,P4HA1,VAPB,CANX,TMBIM6,TMCO1,DEGS1,PDIA6,MANF,ERLIN2,HYOU1,NCSTN,ATP1A1,PPIB,PDIA3,TMED10,PIGG,CALR,BCAP31,APOD,RNF103,EEF1G |
| GOTERM_CC_DIRECT | GO:0042470 | melanosome | 12 | 8.79E-09 | RAB2A,CANX,RAB1A,PDIA6,NCSTN,ATP1A1,RPN1,PPIB,  PDIA3,PDCD6IP,TMED10,BSG |
| GOTERM_CC_DIRECT | GO:0005790 | smooth endoplasmic reticulum | 6 | 6.60E-06 | PRDX4,CANX,HYOU1,PPIB,MYO1D,CALR |
| GOTERM_CC_DIRECT | GO:0071556 | integral component of lumenal side of endoplasmic reticulum membrane | 5 | 2.26E-04 | CD74,CANX,PPL2A,CALR,BCAP31 |
| GOTERM_CC_DIRECT | GO:0016021 | integral component of membrane | 78 | 2.29E-04 | LAMP2,CD74,SEC63,IFNGR1,PLPP1,SLC44A1,ITM2A,APLP2,  TMED2,ERGIC2,SLC15A1,SLC7A8,XBP1,PIGU,SMCHD1,PLP2,TANGO6,SERINC1,KCNJ13,UXS1,THADA,CD58,ADGRL2,  SPCS2,EPCAM,C10orf76,ALG5,VAPB,TM9SF2,PSMA1,  SEC61G,AP3B1,KL,EMC7,IL6ST,STT3A,CD36,YME1L1,SPPL2A,ESYT1,TMBIM6,TSPAN3,SEC11A,TMCO1,DEGS1,FAM171B,  ERLIN2,GPR107,MFSD14A,PEX19,NCSTN,ATP1A1,RPN1,  DNAJB14,FZD6,TMEM67,FUNDC2,SEC11C,SPINT2,LMBRD1,ADAM9,  GPR183,FAXDC2,TMED10,CLSTN1,BSG,ADGRG2,CD34,PIGG,SMIM19,ATP6AP2,PIGP,BCAP31,FAT4,IGF2R,PSAP,TMX2,  RNF103 |
| GOTERM_CC_DIRECT | GO:0005925 | focal adhesion | 14 | 2.55E-04 | RPL23,MSN,GSN,HYOU1,GJA1,NCSTN,PPIB,PDIA3,ADAM9,  PDCD6IP,BSG,LIG4,CALR,IGF2R |
| GOTERM_CC_DIRECT | GO:0005794 | Golgi apparatus | 21 | 7.53E-04 | ITM2A,TMED2,ERGIC2,RAB2A,NUCB1,CPE,VAPB,AP3B1,  CD36,ITM2B,RAB1A,GPR107,GJA1,NCSTN,ATP1A1,UBAP1,TRAPPC11,TMED10,RAB6A,CALR,FAM3C |
| GOTERM_CC_DIRECT | GO:0005829 | cytosol | 52 | 0.002218 | STRAP,SEC63,CTPS2,CAPZB,XPO1,DOPEY1,DNAJA1,RAB18,  XBP1,FBXO7,ST13,MTHFD1,STAG2,EIF3E,BLMH,PRKAR1A  ,DDX6,FBXO3,RPS25,NT5C3A,PRDX4,CSE1L,RPL23,VPS13C,  PSMA1,EIF2S3,SEC61G,GIMAP4,RAB1A,OGT,GSN,FAU,PAN3,GJA1,SEC13,PSMC2,RPL26,PEX19,POLR2H,UBAP1,PDCD6IP,NBEA,PCCA,RAB6A,MYO1D,CALR,PPA1,BCAP31,DPYD,  YTHDF2,DENND1B,EEF1G |
| GOTERM_CC_DIRECT | GO:0005788 | endoplasmic reticulum lumen | 8 | 0.004182 | UGGT2,P4HA1,CANX,PDIA6,HYOU1,PPIB,PDIA3,CALR |
| GOTERM_CC_DIRECT | GO:0033116 | endoplasmic reticulum-Golgi intermediate compartment membrane | 5 | 0.004552 | TMED2,ERGIC2,RAB2A,TMED10,BCAP31 |
| GOTERM_CC_DIRECT | GO:0034663 | endoplasmic reticulum chaperone complex | 3 | 0.005647 | PDIA6,HYOU1,PPIB |
| GOTERM_CC_DIRECT | GO:0005793 | endoplasmic reticulum-Golgi intermediate compartment | 5 | 0.005648 | TMED2,UGGT2,NUCB1,PDIA6,TMED10 |
| GOTERM_CC_DIRECT | GO:0005765 | lysosomal membrane | 9 | 0.008422 | LAMP2,CD74,RAB2A,AP3B1,SPPL2A,NCSTN,LMBRD1,IGF2R,PSAP |
| GOTERM_CC_DIRECT | GO:0000139 | Golgi membrane | 14 | 0.009599 | CD74,DOPEY1,TMED2,RAB2A,VAPB,ITM2B,RAB1A,TMCO1,  GJA1,SEC13,TMED10,CLSTN1,BSG,RAB6A |
| GOTERM_CC_DIRECT | GO:0016324 | apical plasma membrane | 9 | 0.011824 | EPCAM,KL,CD36,MSN,GJA1,ATP1A1,FZD6,ADGRG2,CD34 |
| GOTERM_CC_DIRECT | GO:0043202 | lysosomal lumen | 5 | 0.012247 | LAMP2,CD74,OGN,FUCA1,PSAP |
| GOTERM_CC_DIRECT | GO:0005739 | mitochondrion | 24 | 0.01232 | PDK4,AASS,TTC19,CTPS2,SLC44A1,DNAJA1,FBXO7,MTHFD1,DDX6,UXS1,NT5C3A,P4HA1,PRDX4,YME1L1,SCCPDH,DEGS1,SPATA5,OGT,GJA1,FUNDC2,NUDT9,BSG,BCAP31,PSAP |
| GOTERM_CC_DIRECT | GO:0005615 | extracellular space | 24 | 0.014039 | LAMP2,NUCB1,OGN,LGALS3BP,CPE,SIAE,PRDX4,TPT1,KL,IL6ST,CD36,ITM2B,MANF,MSN,GSN,FAU,PTH,CD109,ADAM9,  CALR,APOD,SPOCK3,IGF2R,PSAP |
| GOTERM_CC_DIRECT | GO:0012507 | ER to Golgi transport vesicle membrane | 4 | 0.017196 | CD74,TMED2,SEC13,TMED10 |
| GOTERM_CC_DIRECT | GO:0000792 | heterochromatin | 3 | 0.020134 | DDX6,SUMO1,SMARCAD1 |
| GOTERM_CC_DIRECT | GO:0030660 | Golgi-associated vesicle membrane | 3 | 0.020134 | ITM2B,SPPL2A,GJA1 |
| GOTERM_CC_DIRECT | GO:0005771 | multivesicular body | 3 | 0.021996 | CD74,TPT1,GJA1 |
| GOTERM_CC_DIRECT | GO:0043209 | myelin sheath | 6 | 0.022036 | CANX,MSN,ATP1A1,PDIA3,PDCD6IP,MYO1D |
| GOTERM_CC_DIRECT | GO:0030176 | integral component of endoplasmic reticulum membrane | 5 | 0.023891 | XBP1,PIGU,ESYT1,TMCO1,PIGG |
| GOTERM_CC_DIRECT | GO:0009986 | cell surface | 12 | 0.02796 | CD74,CD58,EPCAM,CD36,CD109,FZD6,PDIA3,ADAM9,  CLSTN1,ADGRG2,CALR,IGF2R |
| GOTERM_CC_DIRECT | GO:0005764 | lysosome | 7 | 0.031972 | LAMP2,SIAE,PCYOX1,TIAL1,GJA1,CD34,PSAP |
| GOTERM_CC_DIRECT | GO:0043231 | intracellular membrane-bounded organelle | 12 | 0.033508 | AASS,XPO1,TMED2,ERGIC2,DDX6,P4HA1,ITM2B,SPPL2A  ,SEC13,PSMC2,PEX19,ATP1A1 |
| GOTERM_CC_DIRECT | GO:0005769 | early endosome | 7 | 0.033875 | NUCB1,RAB1A,GPR107,GJA1,ZFYVE9,PTP4A2,IGF2R |
| GOTERM_CC_DIRECT | GO:0031012 | extracellular matrix | 8 | 0.037024 | OGN,LGALS3BP,RPS25,RPL23,CANX,RPN1,CALR,SPOCK3 |
| GOTERM_CC_DIRECT | GO:0005887 | integral component of plasma membrane | 23 | 0.040608 | SLC7A2,IFNGR1,PLPP1,SLC15A1,SLC7A8,KCNJ13,CD58,EPCAM,TM9SF2,KL,CD36,TMBIM6,TSPAN3,DEGS1,GJA1,NCSTN,  FZD6,GPR183,BSG,ADGRG2,CD34,BCAP31,IGF2R |
| GOTERM_CC_DIRECT | GO:0016323 | basolateral plasma membrane | 6 | 0.041256 | SLC7A8,EPCAM,MSN,ATP1A1,ADAM9,MYO1D |
| GOTERM_CC_DIRECT | GO:0045177 | apical part of cell | 4 | 0.044184 | CD36,MSN,FZD6,FAT4 |
| GOTERM_CC_DIRECT | GO:0032580 | Golgi cisterna membrane | 4 | 0.045657 | TMED2,NUCB1,UXS1,BCAP31 |
| GOTERM_CC_DIRECT | GO:0000932 | cytoplasmic mRNA processing body | 4 | 0.048675 | DDX6,PAN3,PSMC2,YTHDF2 |
| GOTERM_MF_DIRECT | GO:0051082 | unfolded protein binding | 7 | 0.001002 | DNAJA1,ST13,UGGT2,CANX,TMEM67,PPIB,CALR |
| GOTERM_MF_DIRECT | GO:0044822 | poly(A) RNA binding | 23 | 0.003164 | STRAP,SEC63,YTHDC2,EIF3E,DDX6,SUMO1,RPS25,RPL23,CANX,  TPT1,HNRNPA1,DDX56,MANF,FAU,TIAL1,PAN3,RPL26,RPN1,PPIB,  PDIA3,CALR,YTHDF2,HMGN2 |
| GOTERM_MF_DIRECT | GO:0005515 | protein binding | 110 | 0.003852 | LAMP2,TTC19,CD74,STRAP,CCDC28A,SEC63,IFNGR1,YTHDC2,CTPS2,  ITM2A,XPO1,APLP2,AK6,DNAJA1,TMED2,ERGIC2,SLC7A8,RAB18,XBP1,  FBXO7,ST13,MTHFD1,STAG2,PLP2,UGGT2,RAB2A,EIF3E,NUCB1,OGN,  BLMH,PRKAR1A,DDX6,FBXO3,SERINC1,CLK4,THADA,SUMO1,CD58,  RPS25,EPCAM,CUTC,C10orf76,P4HA1,PRDX4,VAPB,CSE1L,RPL23,CANX,  PSMA1,EIF2S3,SEC61G,TPT1,IL6ST,STT3A,CD36,HNRNPA1,ITM2B,RAB1A,  SPPL2A,ESYT1,TMBIM6,NME7,PDIA6,MSN,OGT,ERLIN2,GSN,ARL14EP,  PAN3,GJA1,UTRN,NUP205,SEC13,ZFYVE9,SSBP3,PSMC2,RPL26,PEX19,  NCSTN,SMARCAD1,ABRAXAS1,ATP1A1,RPN1,FZD6,TMEM67,UBAP1,  PPIB,PDIA3,ADAM9,PDCD6IP,TMED10,CLSTN1,BSG,LIG4,RAB6A,  CALR,ATP6AP2,BCAP31,DPYD,APOD,FAT4,ZNF846,ZNF33B,FAM3C,  IGF2R,PSAP,YTHDF2,HMGN2,RNF103,EEF1G |
| GOTERM_MF_DIRECT | GO:0001540 | beta-amyloid binding | 4 | 0.005176 | CD74,ITM2A,ITM2B,CLSTN1 |
| GOTERM_MF_DIRECT | GO:0019904 | protein domain specific binding | 8 | 0.006048 | LAMP2,XPO1,ST13,DDX6,GSN,ATP1A1,RAB6A,MYO1D |
| GOTERM_MF_DIRECT | GO:0051087 | chaperone binding | 5 | 0.009996 | DNAJA1,ST13,HYOU1,ATP1A1,CALR |
| GOTERM_MF_DIRECT | GO:0031625 | ubiquitin protein ligase binding | 9 | 0.010215 | DNAJA1,XBP1,FBXO7,PRKAR1A,SUMO1,TMBIM6,ERLIN2,FZD6,CALR |
| GOTERM_MF_DIRECT | GO:0098641 | cadherin binding involved in cell-cell adhesion | 9 | 0.010892 | CAPZB,EIF3E,DDX6,EPCAM,VAPB,EIF2S3,RAB1A,BSG,EEF1G |
| GOTERM_MF_DIRECT | GO:0004576 | oligosaccharyl transferase activity | 2 | 0.04083 | ALG5,STT3A |
| KEGG_PATHWAY | hsa04141 | Protein processing in endoplasmic reticulum | 15 | 3.84E-08 | SEC63,DNAJA1,EIF2AK1,XBP1,UGGT2,CANX,SEC61G,STT3A,  PDIA6,HYOU1,SEC13,RPN1,PDIA3,CALR,BCAP31 |
| KEGG_PATHWAY | hsa03060 | Protein export | 5 | 2.15E-04 | SEC63,SPCS2,SEC61G,SEC11A,SEC11C |
| KEGG_PATHWAY | hsa03013 | RNA transport | 8 | 0.007774 | STRAP,XPO1,EIF3E,SUMO1,EIF2S3,NUP205,SEC13,RGPD2 |
| KEGG_PATHWAY | hsa00563 | Glycosylphosphatidylinositol(GPI)-anchor biosynthesis | 3 | 0.043141 | PIGU,PIGG,PIGP |
| KEGG_PATHWAY | hsa00240 | Pyrimidine metabolism | 5 | 0.049459 | CTPS2,NT5C3A,NME7,POLR2H,DPYD |
