# Supplementary material for: Comparative Gene Expression Profiles in Parathyroid Adenoma and Normal Parathyroid Tissue
Source: J Clin Med. 2019 Mar 2;8(3):297. doi: 10.3390/jcm8030297 (PMC6463127; doi:10.3390/jcm8030297)
Supplement: Supplementary file 1 [file jcm-08-00297-s001.zip › Supplemental Table 2. All up- and down-regulated genes.docx]

Supplemental Table 2. Up- and down-regulated genes in parathyroid adenomas compared to normal parathyroid tissues

1. Up-regulated 45 genes

| **Gene Name** | **Fold change** | **T-test Pvalue UEV** | **FDR** |
| --- | --- | --- | --- |
| BMP2K | 5.67 | 5.90.E-08 | 6.39E-05 |
| MED12 | 4.79 | 5.38.E-08 | 6.39E-05 |
| NUFIP1 | 2.47 | 1.33.E-07 | 8.55E-05 |
| KRBOX4 | 3.60 | 2.96.E-07 | 0.00011 |
| ATAD2 | 3.70 | 5.41.E-07 | 0.000156 |
| GPBP1 | 2.85 | 6.49.E-07 | 0.000158 |
| LUC7L | 2.07 | 1.16.E-06 | 0.000176 |
| TCHP | 2.76 | 1.20.E-06 | 0.000176 |
| GOLGA8Q | 2.80 | 2.69.E-06 | 0.00025 |
| CCDC174 | 2.02 | 2.97.E-06 | 0.000262 |
| ZNF674 | 3.63 | 3.09.E-06 | 0.000264 |
| CTTNBP2 | 5.49 | 4.82.E-06 | 0.00035 |
| ARIH2 | 5.14 | 5.16.E-06 | 0.000363 |
| GOLGA8O | 2.71 | 6.00.E-06 | 0.00039 |
| PPM1B | 4.14 | 6.18.E-06 | 0.000392 |
| ZNF605 | 2.94 | 6.92.E-06 | 0.000401 |
| COPS7B | 3.61 | 8.75.E-06 | 0.000451 |
| KMT5A | 3.00 | 9.42.E-06 | 0.000459 |
| NPIPA7 | 2.22 | 9.86.E-06 | 0.000459 |
| SLTM | 2.52 | 9.34.E-06 | 0.000459 |
| NPIPA8 | 2.21 | 1.08.E-05 | 0.000476 |
| BCL9L | 2.75 | 1.16.E-05 | 0.000484 |
| SAFB | 2.06 | 1.37.E-05 | 0.000519 |
| ATXN7L3 | 2.67 | 1.59.E-05 | 0.000555 |
| GOLGA8N | 2.29 | 1.69.E-05 | 0.000556 |
| KIF3A | 2.51 | 1.67.E-05 | 0.000556 |
| MNT | 3.80 | 1.70.E-05 | 0.000556 |
| SAFB2 | 2.52 | 1.75.E-05 | 0.000562 |
| EP400 | 2.73 | 2.22.E-05 | 0.000657 |
| ZNF641 | 3.62 | 2.23.E-05 | 0.000657 |
| BRSK1 | 4.09 | 2.28.E-05 | 0.00066 |
| RFXAP | 4.23 | 2.80.E-05 | 0.000757 |
| SMG7 | 2.12 | 2.85.E-05 | 0.000757 |
| ZNF721 | 2.74 | 2.85.E-05 | 0.000757 |
| CPSF6 | 2.86 | 2.98.E-05 | 0.000771 |
| TBC1D3E | 3.56 | 3.02.E-05 | 0.000775 |
| MUC3A | 13.01 | 3.19.E-05 | 0.000788 |
| NOL4L | 3.00 | 3.14.E-05 | 0.000788 |
| RNR2 | 2.70 | 3.30.E-05 | 0.000805 |
| CXXC1 | 3.61 | 3.34.E-05 | 0.000807 |
| CHERP | 3.36 | 3.87.E-05 | 0.000878 |
| EHMT1 | 2.32 | 4.23.E-05 | 0.000936 |
| PRIMPOL | 2.46 | 4.37.E-05 | 0.000956 |
| ZNF135 | 3.19 | 4.49.E-05 | 0.000971 |
| RBM25 | 2.33 | 4.66.E-05 | 0.000982 |

1. Down-regulated 202 genes

| **GeneName** | **FoldChange** | **TtestPvalueUEV** | **FDR** |
| --- | --- | --- | --- |
| DEGS1 | -6.93 | 1.95.E-08 | 6.39E-05 |
| TMBIM6 | -7.28 | 4.65.E-08 | 6.39E-05 |
| SSBP3 | -7.37 | 6.67.E-08 | 6.39E-05 |
| SNORA74A | -46.50 | 7.37.E-08 | 6.39E-05 |
| DPYD | -5.02 | 9.45.E-08 | 7.02E-05 |
| ALG5 | -15.16 | 1.48.E-07 | 8.55E-05 |
| ZNF552 | -5.20 | 1.96.E-07 | 0.000102 |
| NBEAL1 | -3.90 | 2.46.E-07 | 0.00011 |
| OGN | -18.30 | 2.67.E-07 | 0.00011 |
| CALR | -6.21 | 2.90.E-07 | 0.00011 |
| ZNF33A | -4.17 | 3.83.E-07 | 0.000133 |
| SPDYE16 | -12.79 | 4.24.E-07 | 0.000138 |
| FZD6 | -3.90 | 4.53.E-07 | 0.000139 |
| ATP6AP2 | -21.89 | 5.72.E-07 | 0.000157 |
| PIGG | -3.80 | 6.47.E-07 | 0.000158 |
| UBAP1 | -3.57 | 6.68.E-07 | 0.000158 |
| ST13 | -3.20 | 7.75.E-07 | 0.000175 |
| YTHDC2 | -3.57 | 8.17.E-07 | 0.000176 |
| CPE | -10.34 | 9.18.E-07 | 0.000176 |
| PTH | -6.62 | 9.36.E-07 | 0.000176 |
| TM9SF2 | -9.07 | 9.77.E-07 | 0.000176 |
| TMEM67 | -5.73 | 9.91.E-07 | 0.000176 |
| FAM171B | -3.49 | 1.01.E-06 | 0.000176 |
| CANX | -9.07 | 1.10.E-06 | 0.000176 |
| TIAL1 | -5.89 | 1.15.E-06 | 0.000176 |
| FUCA1 | -7.34 | 1.19.E-06 | 0.000176 |
| TMX2 | -8.78 | 1.21.E-06 | 0.000176 |
| SEC61G | -15.64 | 1.24.E-06 | 0.000176 |
| LMBRD1 | -9.98 | 1.25.E-06 | 0.000176 |
| SERINC1 | -6.41 | 1.35.E-06 | 0.00018 |
| RGPD2 | -4.49 | 1.44.E-06 | 0.00018 |
| MFSD14A | -10.25 | 1.44.E-06 | 0.00018 |
| PCYOX1 | -5.88 | 1.45.E-06 | 0.00018 |
| FUNDC2 | -6.02 | 1.45.E-06 | 0.00018 |
| PSMA1 | -7.04 | 1.58.E-06 | 0.000191 |
| SNORA81 | -25.97 | 1.62.E-06 | 0.000192 |
| TMED10 | -8.18 | 1.71.E-06 | 0.000198 |
| TECR | -5.95 | 1.85.E-06 | 0.000209 |
| SCCPDH | -5.27 | 1.96.E-06 | 0.000214 |
| PTP4A2 | -5.07 | 1.97.E-06 | 0.000214 |
| SMIM19 | -10.45 | 2.03.E-06 | 0.000216 |
| DMXL1 | -4.04 | 2.16.E-06 | 0.000225 |
| CTPS2 | -4.73 | 2.24.E-06 | 0.000228 |
| PDIA6 | -5.66 | 2.35.E-06 | 0.000235 |
| SPATA5 | -5.09 | 2.52.E-06 | 0.000248 |
| PTMA | -3.47 | 2.64.E-06 | 0.00025 |
| FBXO3 | -6.11 | 2.69.E-06 | 0.00025 |
| ZFYVE9 | -4.76 | 2.82.E-06 | 0.000257 |
| LGALS3BP | -5.74 | 3.02.E-06 | 0.000262 |
| ADAM9 | -7.29 | 3.02.E-06 | 0.000262 |
| TTC21B | -4.66 | 3.15.E-06 | 0.000264 |
| KCNJ13 | -4.67 | 3.27.E-06 | 0.00027 |
| PDIA3 | -6.88 | 3.93.E-06 | 0.000319 |
| STAG2 | -3.80 | 4.09.E-06 | 0.000327 |
| DNAJB14 | -2.57 | 4.30.E-06 | 0.000338 |
| DOPEY1 | -3.08 | 4.39.E-06 | 0.000338 |
| AK6 | -5.46 | 4.45.E-06 | 0.000338 |
| VPS13D | -3.91 | 4.54.E-06 | 0.000338 |
| IGF2R | -2.57 | 4.54.E-06 | 0.000338 |
| RAB1A | -4.69 | 4.85.E-06 | 0.00035 |
| POLR2H | -4.03 | 4.96.E-06 | 0.000354 |
| ZNF33B | -4.96 | 5.44.E-06 | 0.000378 |
| FAT4 | -3.52 | 5.62.E-06 | 0.000383 |
| CD109 | -12.55 | 5.66.E-06 | 0.000383 |
| ERGIC2 | -5.23 | 5.96.E-06 | 0.00039 |
| UXS1 | -6.88 | 5.98.E-06 | 0.00039 |
| FGGY | -5.30 | 6.60.E-06 | 0.000401 |
| VPS13B | -6.58 | 6.65.E-06 | 0.000401 |
| YTHDF2 | -3.28 | 6.81.E-06 | 0.000401 |
| IFNGR1 | -3.68 | 6.94.E-06 | 0.000401 |
| SLC7A2 | -4.22 | 6.96.E-06 | 0.000401 |
| SPPL2A | -10.47 | 7.00.E-06 | 0.000401 |
| XBP1 | -3.39 | 7.02.E-06 | 0.000401 |
| CCDC28A | -11.56 | 7.27.E-06 | 0.000411 |
| EPCAM | -11.83 | 7.38.E-06 | 0.000413 |
| SIAE | -9.36 | 7.51.E-06 | 0.000416 |
| APOD | -15.82 | 7.76.E-06 | 0.000423 |
| SPCS2 | -7.45 | 7.88.E-06 | 0.000423 |
| SMCHD1 | -3.78 | 7.88.E-06 | 0.000423 |
| GPR183 | -5.90 | 8.28.E-06 | 0.000436 |
| SLC7A8 | -4.71 | 8.38.E-06 | 0.000436 |
| PDCD6IP | -3.09 | 8.39.E-06 | 0.000436 |
| BEX4 | -6.89 | 9.03.E-06 | 0.000459 |
| NME7 | -5.18 | 9.23.E-06 | 0.000459 |
| ITM2A | -6.59 | 9.29.E-06 | 0.000459 |
| GSN | -5.69 | 9.63.E-06 | 0.000459 |
| FKBP9 | -3.85 | 9.67.E-06 | 0.000459 |
| PSMC2 | -3.60 | 9.78.E-06 | 0.000459 |
| GIMAP4 | -2.36 | 9.85.E-06 | 0.000459 |
| PIGU | -6.47 | 9.87.E-06 | 0.000459 |
| EIF2AK1 | -4.71 | 1.03.E-05 | 0.000475 |
| XPO1 | -4.60 | 1.05.E-05 | 0.000476 |
| ESYT1 | -2.94 | 1.05.E-05 | 0.000476 |
| TTC19 | -3.85 | 1.06.E-05 | 0.000476 |
| HEATR5A | -2.79 | 1.08.E-05 | 0.000476 |
| CD34 | -5.42 | 1.10.E-05 | 0.000482 |
| RAB2A | -5.86 | 1.13.E-05 | 0.000484 |
| SPOCK3 | -27.85 | 1.15.E-05 | 0.000484 |
| ATP1A1 | -14.59 | 1.15.E-05 | 0.000484 |
| SEC11A | -4.77 | 1.15.E-05 | 0.000484 |
| PIGP | -8.94 | 1.16.E-05 | 0.000484 |
| SUMO1 | -6.65 | 1.19.E-05 | 0.000491 |
| FAM3C | -5.69 | 1.21.E-05 | 0.000494 |
| FAXDC2 | -4.45 | 1.23.E-05 | 0.0005 |
| ZNF407 | -2.79 | 1.27.E-05 | 0.000512 |
| CLK4 | -7.40 | 1.31.E-05 | 0.000516 |
| ZNF846 | -2.67 | 1.32.E-05 | 0.000516 |
| NUP205 | -3.65 | 1.32.E-05 | 0.000516 |
| TSPAN3 | -7.10 | 1.33.E-05 | 0.000516 |
| AP3B1 | -3.13 | 1.34.E-05 | 0.000516 |
| KL | -15.08 | 1.34.E-05 | 0.000516 |
| PCCA | -4.13 | 1.36.E-05 | 0.000519 |
| DNAJA1 | -3.83 | 1.40.E-05 | 0.000526 |
| FAU | -4.45 | 1.43.E-05 | 0.000533 |
| CD36 | -10.77 | 1.43.E-05 | 0.000533 |
| APLP2 | -4.95 | 1.45.E-05 | 0.000534 |
| OGT | -3.48 | 1.47.E-05 | 0.000534 |
| TMCO1 | -4.26 | 1.47.E-05 | 0.000534 |
| STT3A | -11.44 | 1.48.E-05 | 0.000534 |
| RNF103 | -4.99 | 1.49.E-05 | 0.000535 |
| IL6ST | -4.90 | 1.57.E-05 | 0.000555 |
| GJA1 | -15.35 | 1.57.E-05 | 0.000555 |
| LIG4 | -4.55 | 1.59.E-05 | 0.000555 |
| RPL26 | -5.16 | 1.61.E-05 | 0.000556 |
| CD58 | -7.84 | 1.61.E-05 | 0.000556 |
| RAB18 | -4.03 | 1.63.E-05 | 0.000556 |
| TPT1 | -3.99 | 1.64.E-05 | 0.000556 |
| HYOU1 | -2.93 | 1.67.E-05 | 0.000556 |
| PRKAR1A | -5.84 | 1.71.E-05 | 0.000556 |
| PPA1 | -3.26 | 1.71.E-05 | 0.000556 |
| MSN | -6.23 | 1.71.E-05 | 0.000556 |
| GPR107 | -3.09 | 1.74.E-05 | 0.000561 |
| SLC44A1 | -6.42 | 1.77.E-05 | 0.000563 |
| MANF | -4.19 | 1.79.E-05 | 0.000568 |
| EIF3E | -6.04 | 1.86.E-05 | 0.000587 |
| ITM2B | -6.97 | 1.88.E-05 | 0.000589 |
| SPINT2 | -4.78 | 1.92.E-05 | 0.000597 |
| RAB6A | -6.15 | 1.93.E-05 | 0.000597 |
| VAPB | -3.66 | 2.00.E-05 | 0.000614 |
| ABRAXAS1 | -4.09 | 2.04.E-05 | 0.000626 |
| STRAP | -8.49 | 2.07.E-05 | 0.000631 |
| DDX56 | -5.82 | 2.10.E-05 | 0.000635 |
| YME1L1 | -3.31 | 2.13.E-05 | 0.000639 |
| TMED2 | -5.11 | 2.22.E-05 | 0.000657 |
| NUCB1 | -3.96 | 2.25.E-05 | 0.000657 |
| CSE1L | -4.66 | 2.26.E-05 | 0.000657 |
| ARL14EP | -6.15 | 2.26.E-05 | 0.000657 |
| NCSTN | -3.15 | 2.31.E-05 | 0.000664 |
| PREX2 | -3.22 | 2.40.E-05 | 0.000687 |
| TANGO6 | -2.05 | 2.54.E-05 | 0.000722 |
| THADA | -4.77 | 2.57.E-05 | 0.000725 |
| RPS25 | -4.59 | 2.63.E-05 | 0.00074 |
| PPIB | -6.60 | 2.65.E-05 | 0.000741 |
| ADGRL2 | -3.69 | 2.68.E-05 | 0.000745 |
| DNAJC8 | -3.30 | 2.70.E-05 | 0.000746 |
| ERLIN2 | -3.89 | 2.76.E-05 | 0.000757 |
| CD74 | -3.96 | 2.78.E-05 | 0.000757 |
| PRDX4 | -8.72 | 2.78.E-05 | 0.000757 |
| NT5C3A | -3.57 | 2.83.E-05 | 0.000757 |
| HMGN2 | -5.78 | 2.84.E-05 | 0.000757 |
| SEC11C | -7.58 | 2.87.E-05 | 0.000759 |
| PLPP1 | -7.23 | 2.92.E-05 | 0.00076 |
| EIF2S3 | -5.47 | 2.92.E-05 | 0.00076 |
| RPL23 | -3.46 | 2.92.E-05 | 0.00076 |
| CLSTN1 | -3.74 | 3.03.E-05 | 0.000775 |
| DDX6 | -4.42 | 3.04.E-05 | 0.000775 |
| MYO1D | -3.50 | 3.12.E-05 | 0.000788 |
| SMARCAD1 | -2.89 | 3.14.E-05 | 0.000788 |
| CATSPER2P1 | -4.55 | 3.19.E-05 | 0.000788 |
| DENND1B | -4.01 | 3.19.E-05 | 0.000788 |
| SEC13 | -3.19 | 3.20.E-05 | 0.000788 |
| SEC63 | -3.01 | 3.27.E-05 | 0.000804 |
| NUDT9 | -3.92 | 3.34.E-05 | 0.000807 |
| SNORD97 | -184.89 | 3.38.E-05 | 0.000813 |
| VPS13C | -4.05 | 3.41.E-05 | 0.000816 |
| SLC15A1 | -10.92 | 3.42.E-05 | 0.000816 |
| ADGRG2 | -5.36 | 3.49.E-05 | 0.000829 |
| BSG | -4.54 | 3.54.E-05 | 0.000837 |
| LAMP2 | -5.20 | 3.59.E-05 | 0.000846 |
| TRAPPC11 | -4.61 | 3.67.E-05 | 0.000859 |
| PDK4 | -6.32 | 3.74.E-05 | 0.000867 |
| BCAP31 | -7.41 | 3.76.E-05 | 0.000867 |
| CAPZB | -4.30 | 3.77.E-05 | 0.000867 |
| UTRN | -3.00 | 3.82.E-05 | 0.000876 |
| BLMH | -4.08 | 3.88.E-05 | 0.000878 |
| PAN3 | -3.89 | 3.90.E-05 | 0.000878 |
| UGGT2 | -5.41 | 3.90.E-05 | 0.000878 |
| CUTC | -5.57 | 4.03.E-05 | 0.000903 |
| HNRNPA1 | -4.04 | 4.05.E-05 | 0.000905 |
| NBEA | -3.35 | 4.37.E-05 | 0.000956 |
| C10orf76 | -5.28 | 4.38.E-05 | 0.000956 |
| PLP2 | -3.91 | 4.48.E-05 | 0.000971 |
| PSAP | -5.57 | 4.51.E-05 | 0.000971 |
| FBXO7 | -3.26 | 4.52.E-05 | 0.000971 |
| ZFX | -2.81 | 4.57.E-05 | 0.000979 |
| RPN1 | -5.37 | 4.59.E-05 | 0.00098 |
| AASS | -2.51 | 4.65.E-05 | 0.000982 |
| EEF1G | -5.40 | 4.66.E-05 | 0.000982 |
| PEX19 | -3.24 | 4.75.E-05 | 0.000991 |
| MTHFD1 | -3.69 | 4.75.E-05 | 0.000991 |
| P4HA1 | -4.75 | 4.78.E-05 | 0.000991 |
| EMC7 | -6.19 | 4.78.E-05 | 0.000991 |
