# Supplementary figures and images for: Comparative Gene Expression Profiles in Parathyroid Adenoma and Normal Parathyroid Tissue
Source: J Clin Med. 2019 Mar 2;8(3):297. doi: 10.3390/jcm8030297 (PMC6463127; doi:10.3390/jcm8030297)

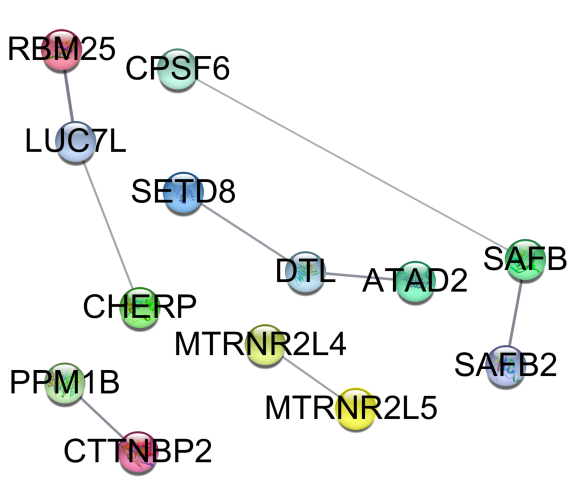

Supplement: Supplementary file 1 [file jcm-08-00297-s001.zip › Supplemental Figure 1.tif]

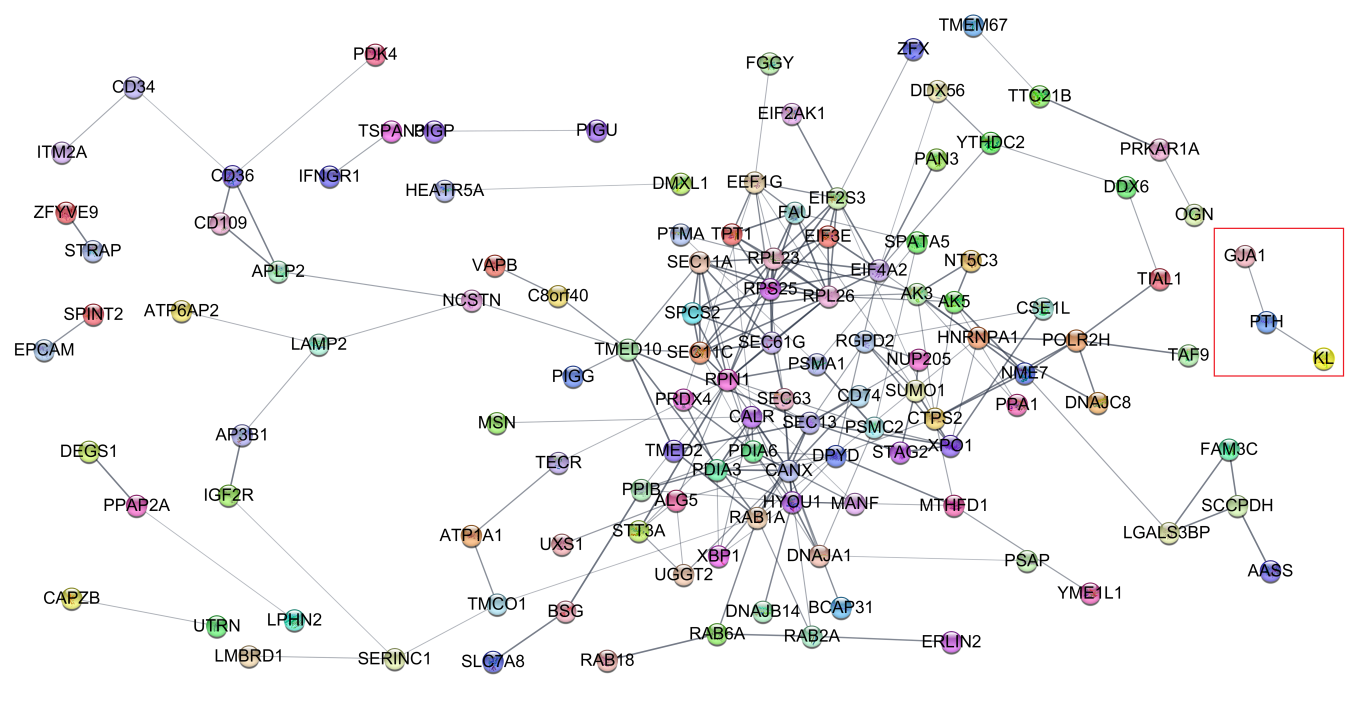

Supplement: Supplementary file 1 [file jcm-08-00297-s001.zip › Supplemental Figure 2.tif]
